# Cerebral palsy and developmental intellectual disability in children younger than 5 years: Findings from the GBD-WHO Rehabilitation Database 2019

**DOI:** 10.3389/fpubh.2022.894546

**Published:** 2022-08-25

**Authors:** Bolajoko O. Olusanya, Melissa Gladstone, Scott M. Wright, Mijna Hadders-Algra, Nem-Yun Boo, M. K. C. Nair, Nihad Almasri, Vijaya Kancherla, Maureen E. Samms-Vaughan, Angelina Kakooza-Mwesige, Tracey Smythe, Christie del Castillo-Hegyi, Ricardo Halpern, Olaf K. de Camargo, Jalal Arabloo, Aziz Eftekhari, Amira Shaheen, Sheffali Gulati, Andrew N. Williams, Jacob O. Olusanya, Donald Wertlieb, Charles R. J. Newton, Adrian C. Davis

**Affiliations:** ^1^Centre for Healthy Start Initiative, Lagos, Nigeria; ^2^Department of Women and Children's Health, Institute of Life Course and Medical Sciences, University of Liverpool, Liverpool, United Kingdom; ^3^Division of General Internal Medicine, Johns Hopkins Bayview Medical Center, Johns Hopkins University School of Medicine, Baltimore, MD, United States; ^4^Division of Developmental Neurology, Department of Paediatrics, University Medical Center Groningen, University of Groningen, Groningen, Netherlands; ^5^Department of Population Medicine, Faculty of Medicine and Health Sciences, Universiti Tunku Abdul Rahman, Selangor, Malaysia; ^6^Child Development Centre, Thiruvananthapuram Medical College, Thiruvananthapuram, Kerala, India; ^7^Department of Physiotherapy, The University of Jordan, Amman, Jordan; ^8^Department of Epidemiology Epidemiologist, Center for Spina Bifida Prevention Rollins School of Public Health, Emory University, Atlanta, GA, United States; ^9^Department of Child and Adolescent Health, The University of the West Indies, Kingston, Jamaica; ^10^Department of Pediatrics and Child Health, Makerere University College of Health Sciences, Kampala, Uganda; ^11^International Centre for Evidence in Disability, London School of Hygiene & Tropical Medicine, London, United Kingdom; ^12^Department of Emergency Medicine, CHI St. Vincent, Little Rock, AR, United States; ^13^Child Development Outpatient Clinic, Hospital da Criança Santo Antônio, Santa Casa de Porto Alegre, Porto Alegre, RS, Brazil; ^14^CanChild Centre for Childhood Disability Research, McMaster University, Hamilton, ON, Canada; ^15^Health Management and Economics Research Center, Health Management Research Institute, Iran University of Medical Sciences, Tehran, Iran; ^16^Department of Toxicology and Pharmacology, Tabriz University of Medical Sciences, Tabriz, Iran; ^17^Division of Public Health, Faculty of Medicine and Health Sciences, An-Najah National University, Nablus, Palestine; ^18^Center of Excellence & Advanced Research on Childhood Neurodevelopmental Disorders, Child Neurology Division, Department of Pediatrics, All India Institute of Medical Sciences, New Delhi, India; ^19^Virtual Academic Unit, Children's Directorate, Northampton General Hospital, Northampton, United Kingdom; ^20^Eliot-Pearson Department of Child Development, Tufts University, Medford, MA, United States; ^21^Kenya Medical Research Institute–Wellcome Trust Research Programme, Centre for Geographic Medicine Research (Coast), Kenya Medical Research Institute, KiIifi, Kenya; ^22^Department of Population Health Sciences, London School of Economics, London, United Kingdom; ^23^Vision and Eye Research Institute, School of Medicine, Anglia Ruskin University, Cambridge, United Kingdom

**Keywords:** cerebral palsy, intellectual disability, rehabilitation, global health, developmental disabilities, global burden of disease, early intervention, SDGs

## Abstract

**Objective:**

Children with developmental disabilities are associated with a high risk of poor school enrollment and educational attainment without timely and appropriate support. Epidemiological data on cerebral palsy and associated comorbidities required for policy intervention in global health are lacking. This paper set out to report the best available evidence on the global and regional prevalence of cerebral palsy (CP) and developmental intellectual disability and the associated “years lived with disability” (YLDs) among children under 5 years of age in 2019.

**Methods:**

We analyzed the collaborative 2019 Rehabilitation Database of the Global Burden of Disease (GBD) Study and World Health Organization for neurological and mental disorders available for 204 countries and territories. Point prevalence and YLDs with 95% uncertainty intervals (UI) are presented.

**Results:**

Globally, 8.1 million (7.1–9.2) or 1.2% of children under 5 years are estimated to have CP with 16.1 million (11.5–21.0) or 2.4% having intellectual disability. Over 98% resided in low-income and middle-income countries (LMICs). CP and intellectual disability accounted for 6.5% and 4.5% of the aggregate YLDs from all causes of adverse health outcomes respectively. African Region recorded the highest prevalence of CP (1.6%) while South-East Asia Region had the highest prevalence of intellectual disability. The top 10 countries accounted for 57.2% of the global prevalence of CP and 62.0% of the global prevalence of intellectual disability.

**Conclusion:**

Based on this Database, CP and intellectual disability are highly prevalent and associated with substantial YLDs among children under 5 years worldwide. Universal early detection and support services are warranted, particularly in LMICs to optimize school readiness for these children toward inclusive education as envisioned by the United Nations' Sustainable Development Goals.

## Introduction

Children under 5 years are widely acknowledged as an important cohort for evaluating the overall health and well-being of any population (1). For several years policymakers have used under-5 mortality as a key indicator of progress in global health and have made targeted reductions in under-5 mortality a central policy objective for global investment in child health (2). The science of human brain development has shown that investments in early childhood, particularly from birth to five years, are the foundation for a prosperous and sustainable society (3, 4). In 2015, the United Nations' Sustainable Development Goals (SDGs) mandated the monitoring of all children under 5 years of age at risk of not realizing their developmental potential to ensure that these children are offered the requisite support services that adequately prepare them for school enrolment (5). However, children with disabilities have a greater risk of poor or sub-optimal development in early childhood compared to children without disabilities (6, 7).

In 2018, the Global Burden of Diseases, Injuries, and Risk Factors Study (GBD) estimated that over 53 million children under 5 years have epilepsy, intellectual disability, hearing loss, vision loss, autism spectrum disorder, or attention deficit hyperactivity disorder (8). Approximately 95% of these children lived in low-income and middle-income countries (LMICs). Although cerebral palsy (CP) is frequently reported as the most common physical disability originating from early childhood (9–11), its exclusion in the GBD 2018 paper was duly acknowledged as a significant limitation (8, 12). To address this omission and provide some indication of the requisite rehabilitation needs, the most recent GBD database produced in collaboration with the World Health Organization (WHO) – the GBD-WHO Rehabilitation Database (labeled as “WHO Rehabilitation Need Estimator”) - now includes data on CP over the life-course (13). This paper sets out to analyze the available global and regional estimates for the prevalence of CP and the associated “years lived with disability” (YLDs). Since developmental intellectual disability (or simply “intellectual disability” hereinafter) is more frequently associated with CP than any other long-term childhood disorder, we also included this condition in this study. The findings will complement our prior reports on the global and regional pattern of developmental disabilities among children under 5 years (8, 14, 15), as well as the recent GBD-related reports on mental and neurological disorders (13, 16).

## Methods

A comprehensive description of the methodology for the GBD-WHO Rehabilitation Database including the underlying modeling strategies has been previously reported (13). As with all GBD papers, the substantive data that formed the basis of this analysis adhered to the Guidelines for Accurate and Transparent Health Estimates Reporting (GATHER), which include recommendations on documentation of data sources, estimation methods, statistical analysis, and statistical code (17). In summary, the point prevalence and YLDs are estimated for 25 health conditions selected by a WHO Expert Panel on Rehabilitation (13). The health conditions are grouped into seven GBD aggregate disease and injury categories: musculoskeletal disorders, neurological disorders, sensory impairments, mental disorders, chronic respiratory diseases, cardiovascular diseases, and neoplasms. CP and intellectual disability are included among the neurological disorders and mental disorders categories, respectively. The estimates for each condition are made for 204 countries and territories categorized into the six WHO regions of Africa, Eastern Mediterranean, European, South-East Asia, The Americas, and Western Pacific (see Appendix 1 in Supplementary material). The high-income countries (HICs) from each region were extracted and grouped into a separate category, based on the World Bank criteria.

Cerebral palsy is a group of neurological disorders that appears in infancy or early childhood and permanently affect body movement and muscle coordination (9, 10). The prevalence of CP was determined indirectly by aggregating all sequelae of neonatal disorders and infectious diseases including preterm birth/low birth weight, neonatal encephalopathy due to birth asphyxia and trauma, neonatal sepsis, and other neonatal infections as well as hemolytic disease and other neonatal jaundice with mention of moderate to severe motor impairment (13, 18). These underlying causes of CP were identified from a systematic review of relevant literature using the International Classification of Diseases, Tenth Revision (ICD-10) codes. Children with mild motor impairment, typically those with ambulation who can walk without help, were excluded in the database on the assumption that they were less likely to require rehabilitation (13).

Intellectual disability is typically defined as a condition of below-average intelligence or mental ability originating before the age of 18 years in line with the Diagnostic and Statistical Manual of Mental Disorders by the American Psychiatric Association (19). The prevalence of intellectual disability (IQ score <70) came from a systematic review of publications since 1990 and included studies that estimated the general population prevalence of intellectual disability (13). Intellectual disability was modeled as an impairment and grouped into five bands based on Intelligence Quotient (IQ) scores, ranging from borderline (70–85), mild (50–69), moderate (35–49), severe (20–34), to profound intellectual disability (0–19). In the GBD Study, an impairment is defined as the sequelae of multiple causes for which better data were available to estimate the overall occurrence than for each underlying cause. Borderline intellectual disability was assumed to be less likely to require rehabilitation and was excluded in computing the prevalence of intellectual disability in the database. A child having both CP and intellectual disability was counted separately for each condition.

Years lived with disability are defined as the years of life lived with any short-term or long-term health loss. YLDs are designed to provide a comparable measure of disease burden across diverse health conditions and impairments rather than a measure of functional status as described in the International Classification of Functioning, Disability and Health (ICF) (20). To calculate YLDs for CP and intellectual disability, the estimated prevalence of each condition at the national, regional, and global level was multiplied by an assigned disability weight based on the severity of the disability. Disability weights are population assessments of the magnitude of health loss associated with specific health outcomes, measured on a scale from 0 to 1, where “0” equals a state of “perfect health” and “1” equals death. For example, the assigned weights for CP vary from 0.01 for mild motor impairment, 0.061 for moderate motor impairment and 0.402 for severe motor impairment based on the degree of ambulation. The disability weights for intellectual disability vary from 0.011 for borderline intellectual functioning to 0.2 for profound intellectual disability based on the degree of difficulty in learning to speak, do simple tasks or follow basic instructions (13). The disability weights were estimated from multi-country population-based surveys using pairwise comparison methods between random pairs of health states as described in detail elsewhere (21).

In general, where there are no primary data, estimates rely on predictive covariates and geographical proximity to countries with data. All computations in the GBD Study were conducted 1,000 times to propagate uncertainty around the estimates for prevalence and YLDs. At every step in the modeling process, the distributions were assessed for sampling error of data inputs, the uncertainty of data corrections for measurement errors, the uncertainty in coefficients from model fit, and the uncertainty of severity distributions and disability weights. Corresponding uncertainty bounds intervals (UIs) for prevalence and YLDs estimates were defined at the 25th and 975th value of 1,000 draws. In this paper, the term “children” refers to children under 5 years of age unless otherwise stated. As this paper is derived from a publicly available database, no ethical approval was required. Estimates are reported along with the 95% uncertainty intervals (UI) in brackets, except stated otherwise.

## Results

Globally, of the 662.8 million children younger than 5 years in 2019, 8.1 million (7.1–9.2) or 1.2 % (1.1–1.4) were estimated to have CP and 16.1 million (11.5–21.0) or 2.4% (1.7–3.2%) had intellectual disability (Table 1). About 53% of children with CP and 54% of children with intellectual disability were male. Most children with CP and intellectual disability resided in LMICs. The estimates for HICs were 359,045 children (326,154–397,121) or 0.6% (0.5–0.6) with CP and 886,977 children (727,734–1,088,596) or 1.4% (1.2–1.8) with intellectual disability. Of the total 27.1 million (19.3–36.1) YLDs among children under 5 years from all causes of fatal and non-fatal health outcomes in 2019, CP accounted for 6.5% or 1.8 million (1.2–2.4) YLDs and intellectual disability accounted for 4.5% or 1.2 million (0.8–1.8) YLDs.

**Table 1:** Global and regional prevalence of cerebral palsy and developmental intellectual disability and the YLDs among children under 5 years in 2019.

**Location**	**Cerebral palsy**				**Developmental intellectual disability**			
	**Prevalence**	**95% uncertainty interval**	**YLDs**	**95% uncertainty interval**	**Prevalence**	**95% uncertainty interval**	**YLDs**	**95% uncertainty interval**
**African region**
Number	2,684,002.9	2,385,081.1–3,033,950.4	586,762.1	408,150.9–793,946.5	3,310,525.9	2,412,002.9–4,238,791.7	261,499.3	169,643.0–375,684.7
Cases per 100,000	1,616.1	1,436.1–1,826.8	353.3	245.8–478.1	1,993.4	1,452.3–2,552.3	157.5	102.1–226.2
**Region of The Americas**
Number	706,407.0	626,193.3–806,022.6	154,178.1	106,230.9–209,656.8	1,189,036.8	978,224.6–1,424,834.2	106,262.7	71,021.0–150,154.6
Cases per 100,000	959.4	850.4–1,094.6	209.4	144.3–284.7	1,614.8	1,328.5–1,935.0	144.3	96.5– 203.9
**East Mediterranean Region**
Number	1,053,861.4	933,962.6–1,202,317.7	229,339.6	157,962.2–315,534.9	2,667,911.1	1,779,861.4–3,585,661.5	186,491.8	115,767.2–275,918.6
Cases per 100,000	1,250.5	1,108.2–1,426.6	272.1	187.4–374.4	3,165.6	2,111.9–4,254.6	221.3	137.4–327.4
**European Region**
Number	435,109.9	396,450.9–480,023.9	95,126.0	65,963.2–128,310.4	926,164.5	686,743.9–1,178,047.5	70,942.1	46,324.9–101,709.5
Cases per 100,000	805.2	733.6–888.3	176.0	122.1–237.4	1,713.9	1,270.8–2,180.0	131.3	85.7–188.2
**South-East Asia Region**
Number	2,357,679.0	2,003,712.7–2,791,591.5	510,862.1	343,125.4–707,366.0	6,317,447.9	4,041,745.9–8,605,559.9	449,331.1	270,088.3–669,597.5
Cases per 100,000	1,427.5	1,213.2–1,690.3	309.3	207.8–428.3	3,825.1	2,447.2–5,210.6	272.1	163.5–405.4
**Western Pacific Region**
Number	927,377.8	772,509.7–1,134,088.2	201,611.8	135,266.0–281,416.5	1,766,440.1	1,416,940.5–2,167,897.7	153,070.6	100,938.6–217,222.6
Cases per 100,000	784.8	653.8–959.8	170.6	114.5–238.2	1,494.9	1,199.2–1,834.7	129.5	85.4– 183.8
**World Bank High-Income**
Number	359,045.2	326,153.6–397,121.3	78,757.3	54,258.9–105,746.3	886,977.2	727,733.8–1,088,595.9	78,097.0	52,323.3–109,403.1
Cases per 100,000	580.3	527.1–641.8	127.3	87.7–170.9	1,433.6	1,176.2–1,759.4	126.2	84.6– 176.8
**Global**
Number	8,071,408.0	7,113,334.0–9,231,577.0	1,757,372.1	1,209,309.2–2,404,752.9	16,057,583.8	11,515,194.1–20,980,652.2	1,222,295.1	782,852.1–1,774,628.7
Cases per 100,000	1,217.7	1,073.2–1,392.7	265.1	182.4–362.8	2,422.5	1,737.2–3,165.3	184.4	118.1–267.7

*YLDs, years lived with disability*.

**Figure 1:**
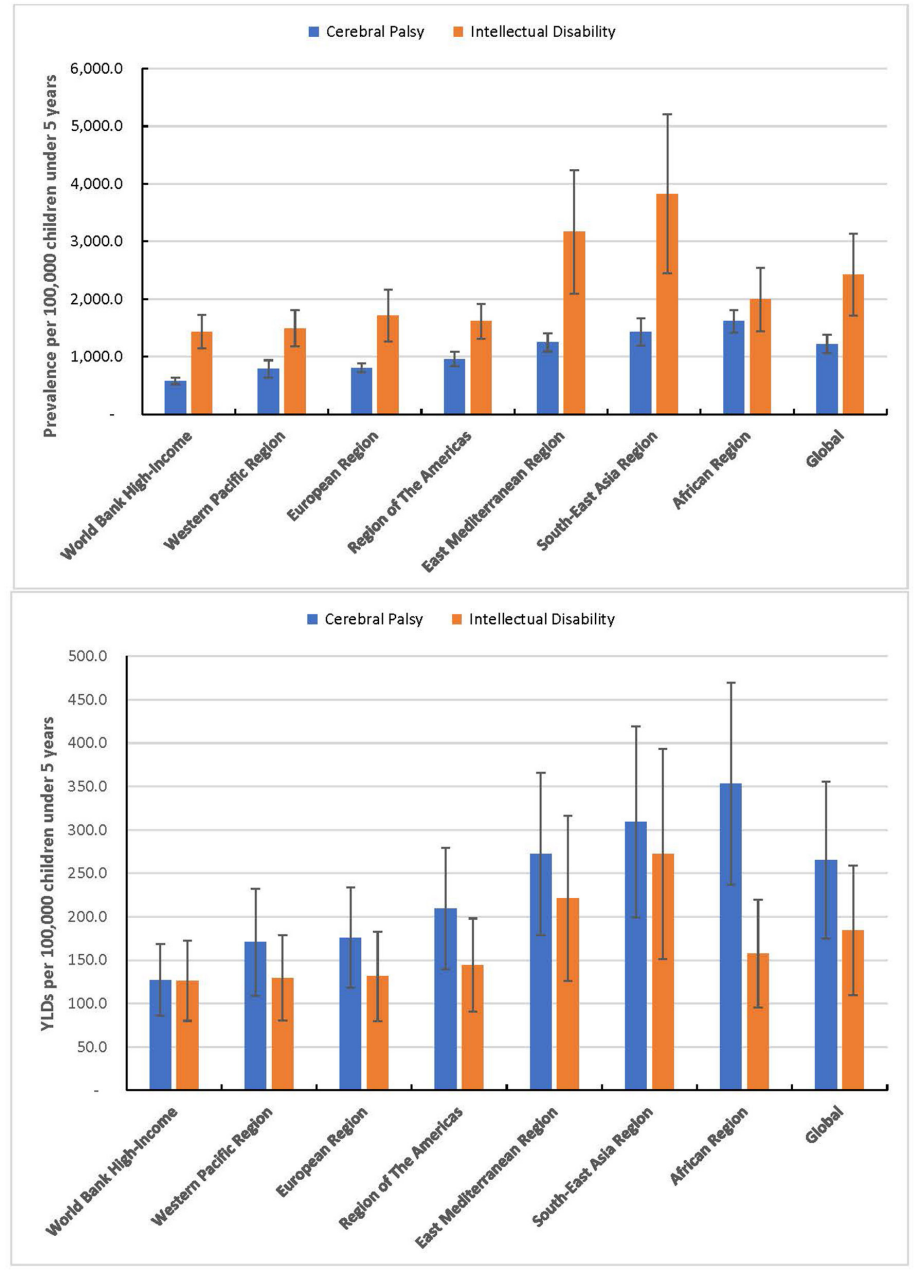
Global and regional prevalence of cerebral palsy and developmental intellectual disability and the YLDs among children under 5 years in 2019.

**Figure 2:**
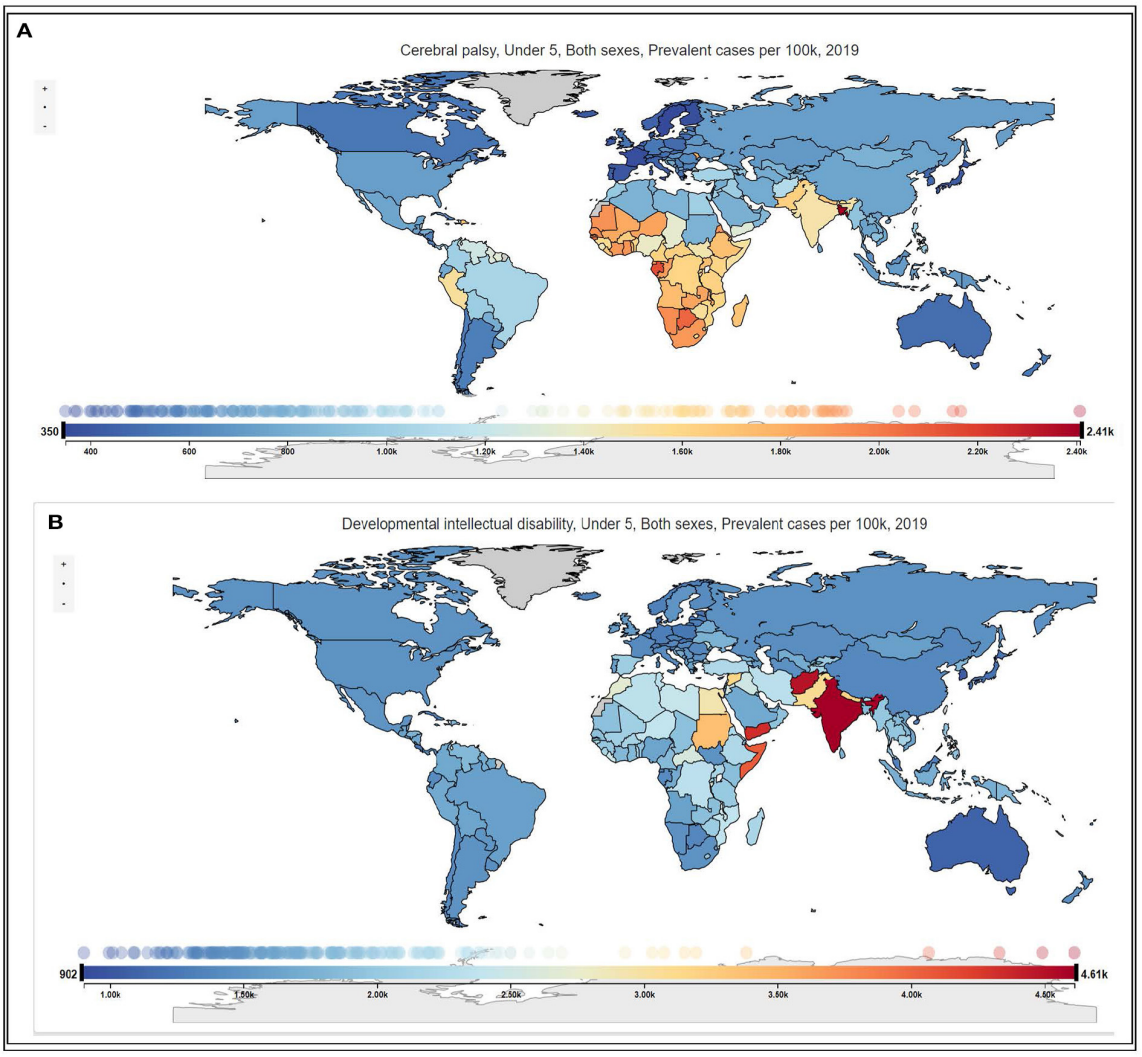
**(A,B)** Global prevalence per 100,000 population of cerebral palsy and developmental intellectual disability among children under 5 years in 2019.

Figure 1 shows that the African Region had the highest prevalence of children with CP of 1.6% (1.4–1.8) or 2.7 million (2.4–3.0) and the highest YLDs of 586,762 (408,151–793,947). South-East Asia Region had the highest prevalence of children with intellectual disability of 3.8% (2.4–5.2) or 6.3 million (4.0–8.6) with an associated YLDs 449,331 (270,088–669,598). The geographical distribution of the prevalence of CP and intellectual disability at country level is presented in Figure 2. India recorded the highest population of children with CP and intellectual disability and the associated YLDs (Table 2). The prevalence of CP and the associated YLDs was highest in Bangladesh, while the prevalence of intellectual disability and the associated YLDs was highest in India. The top 10 countries accounted for 57.2% or 4.6 million of all children with CP and 62.0% or ~10 million of children with intellectual disability globally. These countries also accounted for 57.1% of the global YLDs for CP and 60.4% of the YLDs for intellectual disability. Except for the USA which ranked 10th with the number of children with intellectual disability, the top 10 countries were predominantly LMICs. Among children with CP, 4 (40%) of the top 10 countries with the highest population and 8 (80%) of the top 10 countries with the highest prevalence were from Africa.

**Table 2:** Ten leading countries based on the prevalence of cerebral palsy and developmental intellectual disability and the YLDs among children under 5 years in 2019.

		**Prevalence**	**95% UI**	**Country**	**YLDs**	**95% UI**
**Cerebral palsy**						
**Rank based on No. of cases**	**Country**					
**1**	India	1,741,232.1	1,464,641.9–2,094,260.6	India	376,934.2	251,818.6–525,062.1
**2**	China	617,227.6	510,482.7–763,254.5	China	133,982.7	90,676.3–187,606
**3**	Pakistan	499,455.5	420,258.4–602,818	Pakistan	107,984.6	72,469.9–150,034
**4**	Nigeria	469,079.2	411,845.5–538,426.6	Nigeria	103,274.0	71,648.7–139,313.1
**5**	Bangladesh	330,902.7	283,252.2–389,202.8	Bangladesh	72,104.4	49,300.9–99,638.7
**6**	Ethiopia	286,594.3	248,242.1–333,119.4	Ethiopia	61,847.6	42,509–84,242.6
**7**	Democratic Republic of the Congo	219,682.7	194,823.1–248,569	Democratic Republic of the Congo	48,524.8	32,983.8–66,965.4
**8**	Brazil	162,259.4	143,872.2–183,144.3	Brazil	35,227.4	24,238.1–47,840.6
**9**	United Republic of Tanzania	151,225.0	134,382.6–170,457.2	United Republic of Tanzania	33,121.6	23,190.4–45,329.5
**10**	Indonesia	141,176.9	118,342–169,567.5	Indonesia	30,529.1	20,508.4–42,394.6
**Rank based on Rate/100,000**						
**1**	Bangladesh	2,407.3	2,060.6–2,831.4	Bangladesh	524.6	358.7–724.9
**2**	Comoros	2,166.6	1,921.2–2,445.9	Comoros	469.4	318.9–646.6
**3**	Gabon	2,150.9	1,944.7–2,387.8	Gabon	467.4	317.8–640.8
**4**	Botswana	2,072.9	1,866.5–2,321.2	Botswana	449.9	309.5–609.9
**5**	Guinea-Bissau	2,040.7	1,811.9–2,283.4	Guinea-Bissau	446.4	309.6–599.8
**6**	Namibia	1,934.8	1,746.1–2,163.4	Namibia	418.2	286.9–569.1
**7**	Gambia	1,926.5	1,715.7–2,162.5	Gambia	416.4	284.8–564.8
**8**	Mauritania	1,915.3	1,706.2–2,149	Senegal	415.8	284.9–566.8
**9**	Senegal	1,912.4	1,710–2,146.6	Mauritania	415.6	288–561.5
**10**	Ghana	1,905.4	1,671.2–2,152.9	Ghana	415.0	283.6–567.4
**Developmental intellectual disability**						
**Rank based on No. of cases**	**Country**					
**1**	India	5,398,051.8	3,375,173.5–7,453,873	India	374,294.2	224,952.6–564,039.1
**2**	China	1,136,764.1	938,177.4–1,375,414.6	China	101,402.4	66,371.4–142,134.1
**3**	Pakistan	938,551.6	613,785.6–1,269,948.7	Pakistan	66,873.9	41,700.6–99,348.1
**4**	Nigeria	548,985.2	435,326.9–685,929	Nigeria	47,227.3	31,529.5–67,219.2
**5**	Ethiopia	370,801.6	260,947.9–486,018.4	Indonesia	28,322.2	18,743.2–40,323.8
**6**	Indonesia	342,570.7	257,462.2–435,046.4	Ethiopia	27,446.9	17,640.7–40,004.4
**7**	Democratic Republic of the Congo	317,774.3	208,151.5–426,136.3	United States of America	24,738.7	16,133–35,132.4
**8**	Egypt	316,966.1	215,476.6–417,973	Democratic Republic of the Congo	23,174.0	14,565.5–34,256.6
**9**	Afghanistan	298,448.8	190,228.1–406,802.4	Brazil	22,544.6	15,014.2–31,668.2
**10**	United States of America	282,629.5	223,324.4–355,956.7	Bangladesh	22,412.2	14,477.1–32,482.1
**Rank based on Rate/100,000**						
**1**	India	4,610.9	2,883–6,367	India	319.7	192.2–481.8
**2**	Afghanistan	4,491.2	2,862.7–6,121.8	Afghanistan	303.6	183.9–462
**3**	Yemen	4,330.0	2,773.8–5,853.4	Yemen	284.4	169.4–432.2
**4**	Somalia	4,065.9	2,552.6–5,572.1	Somalia	279.0	170.6–425.4
**5**	Sudan	3,382.1	2,176.1–4,623.7	Sudan	233.2	142–353.2
**6**	Nepal	3,194.5	2,100.9–4,296.7	Nepal	230.7	143.5–340.8
**7**	Syrian Arab Republic	3,151.5	2,087.9–4,262.1	Pakistan	219.1	136.6–325.4
**8**	Pakistan	3,074.5	2,010.7–4,160.2	Syrian Arab Republic	216.8	133.3–322.5
**9**	Palestine	3,030.1	2,006.4–4,053.8	Palestine	206.4	125.7–308.3
**10**	Egypt	2,928.0	1,990.5–3,861.1	Republic of Moldova	205.7	136.4–294.1

*YLDs, years lived with disability; UI, uncertainty interval*.

## Discussion

The dearth of population-based data for specific health conditions from birth across many nations, especially in LMICs, has resulted in a growing reliance on statistical estimation of health outcomes as a surrogate for guiding global health policies and interventions (22). Conceptual and operational challenges in measuring disabilities among children in different cultural contexts at the population-level persist (23, 24). The GBD modeling efforts thus offer an invaluable undertaking in the epidemiology of developmental disorders for global policy intervention. Unlike prior reports, the GBD-WHO collaboration provides an additional layer of quality control for the conventional GBD database through subject expert consultations. Arguably, the estimates reported in this study represent the best available global estimates of children under 5 years with CP and intellectual disability. The findings clearly establish that these conditions are highly prevalent worldwide with LMICs accounting for the greatest burden (i.e., prevalence and YLDs). They also underscore the necessity for primary prevention initiatives and provide independent estimates of the magnitude of the rehabilitation needs for these conditions within the integrated health care systems envisaged by WHO (13).

The global estimate of 1.2% or 12 per 1,000 children under 5 years for CP in this study represents 16.2% of the estimated 50 million of all children and adults with CP (13). This estimate is higher than those in several epidemiological studies which report a global prevalence of between 1 and 4 per 1,000 live births or 1,000 children of all ages (9, 10, 25). However, the global estimates in the literature are almost entirely derived from studies conducted in high-income countries. The GBD-WHO estimate of 0.6% or 5.8 per 1,000 children under 5 years for all high-income countries is higher than the reported estimates for this age group (26, 27). For example, the prevalence of CP among children aged 3–5 years in USA using parental household surveys is estimated at 0.3% or 2.8 per 1,000 (26). However, this estimate is likely to be higher if the younger children below the age of 3 years were included in the estimate. The disproportionately higher prevalence of adverse perinatal and neonatal conditions in LMICs, particularly in sub-Saharan Africa and South Asia, would also suggest higher estimates than reported in the few available population-based studies (9, 28, 29). A detailed comparative analysis with estimates from systematic reviews is difficult principally due to marked variations in methodology, mean age at diagnosis, the age-group of participants, choice of denominator (birth vs. period prevalence) in the underlying studies and the dearth of studies from many regions especially in LMICs (29, 30). In addition, the age range of the children reported across studies varied considerably and the specific prevalence of these conditions among all children under 5 years is seldom reported. In LMICs, children younger than 2 years are commonly excluded because of the view that these conditions are too difficult to detect at this age especially in routine population-based household surveys. Reports also suggest that less than half of children are clinically diagnosed by 24 months of age compared to the practice in HICs (9). Moreover, many children with CP die before their second birthday, thus remain undiagnosed and many may not be counted at all (31). There is a growing international recognition of the technological advances to make early detection and intervention for CP before the age of 2 years feasible even in countries where clinical diagnosis may typically be delayed until age 4 years (11, 32). Considering the gross under-ascertainment of cases in LMICs, we hold the view that the true global prevalence of CP among children under 5 years possibly lie between the GBD-WHO estimate and reported estimates in the literature from household surveys.

The global estimates for intellectual disability appear to agree with those reported by Maulik et al. in which the global prevalence among children and adolescents was shown as 1.8% (95% CI: 1.5–2.1) (33), considering that the prevalence of intellectual disability is highest in early childhood and declines thereafter among older children. The reported prevalence for HICs also appears plausible compared to the ~1.6% for children and adolescents in the USA (34). The significantly higher global prevalence of intellectual disability compared to CP in our study is consistent with evidence in the literature principally due to the wider range of comorbidities associated with the former (33–35). Moreover, 1 in 2 children with CP are frequently diagnosed with comorbid intellectual disability (11). The higher estimates of these conditions in LMICs compared with HICs is also in line with previous findings in the literature (9, 10, 33, 36–38), as well as studies among children from low-income households in HICs (35). The substantial YLDs associated with CP and intellectual disability further underscore the need for global initiatives to address these conditions promptly and appropriately when intervention outcomes can be optimized to enhance the opportunities for inclusive formal education as envisaged by the SDGs (5). The higher YLDs associated with CP compared to intellectual disability perhaps reflect the magnitude and scope of the rehabilitation and other support services required by the affected children in early childhood (11, 32).

The disproportionately high prevalence of CP in Africa may be attributable to a constellation of factors which includes the low quality of maternal and child health services and the high proportion of deliveries not attended by skilled health workers. Clinical factors such as birth asphyxia, kernicterus, and neonatal infections have also been implicated as contributors to the high prevalence (36), in contrast to the more common causes like prematurity and low birth weight in HICs (25). Africa's leading contribution to the global burden of CP accords with our earlier report that demonstrated the significantly higher and rising burden of developmental disabilities in Africa among the growing beneficiaries of the highly successful global investments in reducing under-5 mortality since 1990 (14). The leading contribution of Southeast Asia to the global prevalence of intellectual disability appears to be supported by perhaps the most robust nationally representative study on developmental disabilities in India in which the prevalence of CP and intellectual disability in children aged 2 to <6 years was reported as 2.1% (95% CI: 1.3–3.4) and 3.1% (95% CI: 2.2–4.2), respectively (37). The leading risk factors reported for these conditions and other developmental disabilities in India were non-institutionalized delivery, history of perinatal asphyxia, history of neonatal illness, and postnatal neurological (brain) infections. However, the causal factors in about half of the children with intellectual disability are likely to be unknown (33).

It is noteworthy that the countries with the highest population of children with these conditions were not necessarily those with the highest prevalence. Furthermore, the burden of these conditions is highest in the regions of the world that are poorly resourced to provide the requisite support services for these children and their families (9, 38, 39). Consequently, children with these conditions in LMICs are more likely to experience a lower quality of life compared to their peers in HICs (40). These children are also at greater risk of premature mortality (31). While primary prevention should be prioritized, the substantial unmet rehabilitation needs of these children and others with developmental disabilities in LMICs as highlighted by a recent WHO report require urgent and priority attention for these children and their families (39). Whereas the period of interest for early detection and intervention services varies from conception to age 8 years in the literature, it is pertinent to clarify that the focus on the first 5 years of life from birth in this paper is consistent with the most widely recommended clinical framework for the effective management of children with developmental disabilities for school readiness (41). The evidence in this report reinforces our earlier call for a decisive, appropriate, timely and well-coordinated policy intervention to support these children and others with developmental disabilities to place them on the trajectory for school readiness for inclusive education as envisaged under the Sustainable Development Goals (5, 42).

### Limitations

Modeling techniques and the use of proxy measures to generate evidence are now common in highlighting the public and global health importance of health conditions that are currently constrained by the lack of adequate and reliable population-based data. This approach is premised on the principle that the absence of ideal data is not evidence of absence of a health condition that truly warrants policy intervention. However, this approach is not without shortcomings. The limitations frequently associated with GBD methodologies have been extensively reported in accordance with the GATHER guidelines (12, 13, 15, 16, 18). Additionally, despite the continuous efforts toward improving the GBD methodology, the current practice of estimating the prevalence of disabilities based on sequelae of underlying health conditions or surrogates is not without drawbacks. For example, on the one hand, the exclusive use of motor deficits for CP is likely to have resulted in over-estimation of its prevalence because not all motor impairments constitute CP. On the other hand, this approach may have under-estimated children with CP who have a wide range of levels of functioning and those with milder difficulties with functioning without moderate or severe motor manifestations (43). It is reported that GBD plans to estimate the prevalence of CP based on a meta-analysis of available data from registries and cohort studies which would provide more insights on any variance attributable to the GBD methodology and the required adjustments in model parameters (13). The GBD estimates for disabilities still do not fully reflect the complex and dynamic relationship between health conditions and contextual personal or environmental factors as envisaged under the ICF, as such they provide a limited picture of disability. In fact, the threshold for rehabilitation which excluded children with mild motor impairments and borderline intellectual disability as well as the sole use of IQ tests may inadvertently exclude children with functional limitations that require intervention. Another limitation is the wide uncertainty around the estimates for YLDs due to the determination of disability weights (8, 13, 15, 18, 21). Disability weights in GBD Study reflect the severity of a disease and are needed to quantify health losses relating to non-fatal outcomes. However, cultural, educational, environmental, and demographic differences across populations impede the standardization and global comparison of disability weights. Also, disability weights specifically for childhood conditions are still not available. Several ongoing studies on disability weight in different countries are expected to provide further insights on this subject in the future (13). Finally, it was difficult to combine the findings in this study with our earlier reports on developmental disabilities (8, 15), which may be achieved in the future with further improvements in accounting for children with multiple disabilities across multiple developmental domains. Despite these shortcomings, the difficulties in counting and monitoring developmental disabilities routinely through traditional systematic reviews and meta-analyses, household surveys and population-based surveillance programs, particularly in LMICs, means that estimates from statistical modeling remain an invaluable source of data to inform policies and interventions in global health (22).

## Conclusion

Evidence from the 2019 GBD-WHO Rehabilitation Database suggests that CP and intellectual disability are highly prevalent and associated with substantial YLDs among children under 5 years globally. The burden of these conditions, as with other previously reported developmental disabilities, is higher in LMICs where very limited support services exist compared to HICs. Early detection and high-quality rehabilitation programs for the affected children must be prioritized globally. The SDGs provide unprecedented opportunity to develop and promote requisite policies and programs to ensure that the affected children are offered the best possible prospects for optimal development and inclusive education. While these estimates represent the best available data for policymakers in global health, evidence from surveillance registries and household surveys suggest that further research is warranted to determine improved estimates for these conditions especially in regions with hardly any primary data sources.

## Data availability statement

The original contributions presented in the study are included in the article/Supplementary material, further inquiries can be directed to the corresponding author.

## Author contributions

BO conceived the study. BO and MG wrote the first draft, and finalized the manuscript for submission. AD and JO coordinated the data analysis and interpretation with IHME. SW, MH-A, MG, and N-YB critically reviewed the first draft for important intellectual content and provided overall guidance for the revisions. MN, NA, VK, MS-V, AK-M, TS, CC-H, RH, OC, JA, AE, AS, SG, AW, DW, and CN critically reviewed the first draft for important intellectual content. SW, MH-A, N-YB, and CN provided additional critical review of the final draft. All authors read and approved the final version for submission.

## Funding

The substantive data that formed the basis of this article was funded by the Bill & Melinda Gates Foundation.

## Conflict of interest

The authors declare that the research was conducted in the absence of any commercial or financial relationships that could be construed as a potential conflict of interest.

## Publisher's note

All claims expressed in this article are solely those of the authors and do not necessarily represent those of their affiliated organizations, or those of the publisher, the editors and the reviewers. Any product that may be evaluated in this article, or claim that may be made by its manufacturer, is not guaranteed or endorsed by the publisher.

## Supplementary material

Supplementary Appendix 1 Country grouping by the World Health Organization and the World Bank.
