# Supplementary material for: Cerebral palsy and developmental intellectual disability in children younger than 5 years: Findings from the GBD-WHO Rehabilitation Database 2019
Source: Front Public Health. 2022 Aug 25;10:894546. doi: 10.3389/fpubh.2022.894546 (PMC9452822; doi:10.3389/fpubh.2022.894546)
Supplement: Supplementary Appendix 1 — Country grouping by the World Health Organization and the World Bank. [file Data_Sheet_1.PDF]

Appendix 1

WHO regional groupings*

WHO African Region: Algeria, Angola, Benin, Botswana, Burkina Faso, Burundi, Cabo Verde, Cameroon, Central African Republic, Chad, Comoros, Congo, Côte d'Ivoire, Democratic Republic of the Congo, Equatorial Guinea, Eritrea, Ethiopia, Gabon, Gambia, Ghana, Guinea, Guinea-Bissau, Kenya, Lesotho, Liberia, Madagascar, Malawi, Mali, Mauritania, Mauritius, Mozambique, Namibia, Niger, Nigeria, Rwanda, Sao Tome and Principe, Senegal, Seychelles, Sierra Leone, South Africa, South Sudan, Swaziland, Togo, Uganda, United Republic of Tanzania, Zambia, Zimbabwe.

WHO Region of the Americas: Antigua and Barbuda, Argentina, Bahamas, Barbados, Belize, Bolivia (Plurinational State of), Brazil, Canada, Chile, Colombia, Costa Rica, Cuba, Dominica, Dominican Republic, Ecuador, El Salvador, Grenada, Guatemala, Guyana, Haiti, Honduras, Jamaica, Mexico, Nicaragua, Panama, Paraguay, Peru, Saint Kitts and Nevis, Saint Lucia, Saint Vincent and the Grenadines, Suriname, Trinidad and Tobago, the United States of America, Uruguay, Venezuela (Bolivarian Republic of).

WHO South-East Asia Region: Bangladesh, Bhutan, Democratic People's Republic of Korea, India, Indonesia, Maldives, Myanmar, Nepal, Sri Lanka, Thailand, Timor-Leste.

WHO European Region: Albania, Andorra, Armenia, Austria, Azerbaijan, Belarus, Belgium, Bosnia and Herzegovina, Bulgaria, Croatia, Cyprus, Czechia, Denmark, Estonia, Finland, France, Georgia, Germany, Greece, Hungary, Iceland, Ireland, Israel, Italy, Kazakhstan, Kyrgyzstan, Latvia, Lithuania, Luxembourg, Malta, Monaco, Montenegro, Netherlands, Norway, Poland, Portugal, Republic of Moldova, Romania, Russian Federation, San Marino, Serbia, Slovakia, Slovenia, Spain, Sweden, Switzerland, Tajikistan, The former Yugoslav Republic of Macedonia, Turkey, Turkmenistan, Ukraine, the United Kingdom, Uzbekistan.

WHO Eastern Mediterranean Region: Afghanistan, Bahrain, Djibouti, Egypt, Iran (Islamic Republic of), Iraq, Jordan, Kuwait, Lebanon, Libya, Morocco, Oman, Pakistan, Palestine, Qatar, Saudi Arabia, Somalia, Sudan, Syrian Arab Republic, Tunisia, United Arab Emirates, Yemen.

WHO Western Pacific Region: Australia, Brunei Darussalam, Cambodia, China, Cook Islands, Fiji, Japan, Kiribati, Lao People's Democratic Republic, Malaysia, Marshall Islands, Micronesia (Federated States of), Mongolia, Nauru, New Zealand, Niue, Palau, Papua New Guinea, Philippines, Republic of Korea, Samoa, Singapore, Solomon Islands, Tonga, Tuvalu, Vanuatu, Viet Nam.

* All income levels

World Bank High Income Countries

Andorra, Antigua and Barbuda, Aruba, Australia, Austria, Bahamas, The Bahrain, Barbados, Belgium, Bermuda, British Virgin Islands, Brunei Darussalam, Canada, Cayman Islands, Channel Islands, Chile, Croatia, Curacao, Cyprus, Czech Republic, Denmark, Estonia, Faroe Islands, Finland, France, French Polynesia, Germany, Gibraltar, Greece, Greenland, Guam, Hong Kong Sar, China, Hungary, Iceland, Ireland, Isle of Man, Israel, Italy, Japan, Korea, Rep., Kuwait, Latvia, Liechtenstein, Lithuania, Luxembourg, Macao Sar, China, Malta, Monaco, Nauru, Netherlands, New Caledonia, New Zealand, Northern Mariana Islands, Norway, Oman, Palau, Poland, Portugal, Puerto Rico, Qatar, San Marino, Saudi Arabia, Seychelles, Singapore, Sint Maarten (Dutch Part), Slovak Republic, Slovenia, Spain, St. Kitts and Nevis, St. Martin (French Part), Sweden, Switzerland, Trinidad and Tobago, Turks and Caicos Islands, United Arab Emirates, United Kingdom, United States, Uruguay, Virgin Islands (U.S.).
